# Eugenics No Joke

**Published:** 1914-05

**Authors:** 


### Abstracts and Selections.

### Eugenics No Joke.

It is gratifying indeed to those who have gone deeper into the study of eugenics than the eminent Western sociologist, who recently received so much publicity by his harsh attack on eugenic marriages, to know that the newspapers of the country appear not to have been misled in the matter. The Providence, R. I., *Tribune* of recent date says:

"Many other persons perhaps share the opinion of the California sociologist, who calls eugenics an unqualified joke, without any serious merit to commend it, but their skepticism does not necessary prove any lack of worthiness in the science. It is the ludicrous claims made for it, the ridiculous and often repulsive activity of 'candidates for eugenic marriages' and the ill-advised conduct of self-constituted apostles of the gospel of racial betterment, that have disgusted many conservative and right-thinking people and caused them to believe that eugenics is only a fad which has nothing to commend it to serious consideration.

"As a matter of fact, eugenics is being very widely and very successfully practiced in various ways which are not recognized under its rightful but much-abused title. The love of the spectacular, the dramatic and the daring has given the science the glamor of repellant similarity to sex hygiene and allied subjects which cater to the passion of the bizarre and the erotic. Instead of being advanced as a serious science, holding within itself the potentiality of race improvement, it is dragged down to the level of coarse humor.

"In its true aspect it has made notable progress among the lines indicated by its name. Eugenics may be paraphrased with a fair degree of accuracy as the science of causing children to be 'born well,' of causing them to be cared for properly after they are born and of helping nature to solve more speedily the evolutionary problems to which the world can not devote the time at nature's disposal.

"That is, whatever makes nature stronger, whatever corrects weaknesses, whatever removes dangers, whatever increases the chances for human beings to grow up to lives of usefulness and honor, whatever give them a fair start in the struggle of life—whatever does this, though it may not come strictly within the scientific niceties of the little meaning of the word, is not very far from eugenics, either in principle or in effect.

"There is infinitely more to eugenics than freak laws considering candidates for matrimony guilty of unfitness until they prove their innocence; there is infinitely more to it than sensation-mongers and the makers of cynical, scintillant epigrams. It can not be sneered or laughed away. It can not be waived. It is here. It has been here from the beginning and it will stay to the end. It is one of the great verities which jesters may deride and academic sociologists may denounce and faddists may make absurd without lessening either its potency or its permanency.

"Better housing, better sanitation, better environment for the young, better surroundings for mothers, a higher standard of parenthood, juvenile courts, improved school facilities, protection for the morals of boys and girls, the conservation of childhood—everything that takes hold of the parent and does not let go till the child is himself a parent, belongs in the domain of eugenics. It is arrant nonsense to say that physical and moral standards can not be raised with intelligent supervision, and it only less foolish to control that this can not be done without reducing the science to the level of stock raising. There are many activities, set in motion years ago, and operating with increasing momentum from year to year, which are proving the theories, or, rather, the facts, upon which the science of eugenics is based.

"Possibly the day will come, if the science has a fair chance and plenty of time, when we shall produce a better breed of university sociologists."

